# Biomarkers in Medicines Development—From Discovery to Regulatory Qualification and Beyond

**DOI:** 10.3389/fmed.2022.878942

**Published:** 2022-04-26

**Authors:** Natalie M. Hendrikse, Jordi Llinares Garcia, Thorsten Vetter, Anthony J. Humphreys, Falk Ehmann

**Affiliations:** ^1^Regulatory Science and Innovation Task Force, European Medicines Agency, Amsterdam, Netherlands; ^2^Scientific Advice Office, European Medicines Agency, Amsterdam, Netherlands

**Keywords:** biomarkers, biomarker qualification, Qualification of Novel Methodologies, regulatory science, European Medicines Agency, Innovation Task Force

## Abstract

Biomarkers are important tools in medicines development and clinical practice. Besides their use in clinical trials, such as for enrichment of patients, monitoring safety or response to treatment, biomarkers are a cornerstone of precision medicine. The European Medicines Agency (EMA) emphasised the importance of the discovery, qualification, and use of biomarkers in their Regulatory Science Strategy to 2025, which included the recommendation to enhance early engagement with biomarker developers to facilitate regulatory qualification. This study explores the journey of biomarkers through the EU regulatory system and beyond, based on a review of interactions between developers and the EMA from 2008 to 2020, as well as the use of qualified biomarkers in clinical trials. Of applicants that used early interaction platforms such as the Innovation Task Force, less than half engaged in fee-related follow-up procedures. Results showed that, as compared to companies, consortia were more likely to opt for the Qualification of Novel Methodologies procedure and engage in follow-up procedures. Our results highlight the importance of early engagement with regulators for achieving biomarker qualification, including pre-submission discussions in the context of the qualification procedure. A review of clinical trials showed that all qualified biomarkers are used in practice, although not always according to the endorsed context of use. Overall, this study highlights important aspects of biomarker qualification, including opportunities to improve the seamless support for developers by EMA. The use of qualified biomarkers in clinical trials underlines the importance of regulatory qualification, which will further enable precision medicine for the benefit of patients.

## Introduction

Biomarkers are powerful tools that can serve many purposes in medicines development and clinical practice (1). Examples include the selection of patients and enrichment of study populations for clinical trials, monitoring safety or response to treatment during trials, but also supporting decision-making in the context of precision medicine. In 2001, the Biomarker Definitions Working Group proposed that a biomarker could be defined as “*a characteristic that is objectively measured and evaluated as an indicator of normal biological processes, pathogenic processes, or pharmacologic responses to a therapeutic intervention*” (2). This definition was further elaborated in the Biomarkers, EndpointS, and other Tools (BEST) resource, in which subcategories were defined that reflect different biomarker functions (3, 4). The crucial role of biomarkers in drug development has long been recognised and supported by regulators, who have established frameworks for review of biomarker validation plans and/or data, which may result in regulatory qualification (5). In 2007, a joint pilot procedure by the European Medicines Agency (EMA) and the U.S. Food and Drug Administration (FDA) concerning a panel of nephrotoxicity biomarkers marked the beginning of regulatory qualification of biomarkers in the EU (6, 7). Recently, the EMA underlined the importance of the discovery, qualification and use of biomarkers in their “Regulatory Science Strategy to 2025” (8, 9). One of the primary strategic goals related to regulatory science for human medicines focuses on the integration of science and technology in medicines development, including support of developments in the fields of precision medicine and biomarkers. “*Enhancing early engagement with novel biomarker developers to facilitate regulatory qualification*” is mentioned as a key step to achieve this goal, accompanied by the recommendation to “*critically review the EMA’s biomarker validation process, including duration and opportunities to discuss validation strategies in advance, in order to encourage greater uptake and use.*” Encouraged by these objectives, we aimed to assess the past and current situation, by reviewing the interactions between developers and regulators at various stages of the process leading up to regulatory biomarker qualification. Moreover, our aim was to assess the impact of this regulatory “stamp of approval,” in terms of uptake by the scientific community as well as use in clinical trials.

**FIGURE 1:**
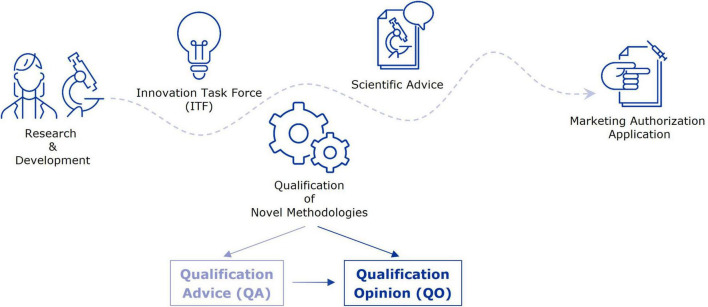
From early-stage research to marketing authorisation–EMA support mechanisms for evidence generation strategies. This figure highlights a selection of interaction platforms at EMA through which applicants can receive guidance and feedback on their evidence generation strategy towards a marketing authorisation application.

At the EMA, the Innovation Task Force (ITF) acts as a first point of contact for developers in early-stage projects with innovative aspects for drug development (10). Interactions take place in the form of informal briefing meetings between applicants and experts from the EU network, and address mainly strategic aspects of regulatory, scientific, and legal nature. A primary goal of the ITF is to fill the gap between early-stage research, performed by academic groups or small to medium-sized companies, but also large companies, and formal regulatory procedures that involve fees, such as Scientific Advice (SA) and the Qualification of Novel Methodologies (QoNM) (Figure 1) (11). The former is an interaction platform at the EMA, where medicine developers can discuss strategies to generate robust evidence for the benefit-risk assessment during the marketing authorisation application (MAA) (12). Biomarkers may constitute an essential part of this strategy and are therefore a common topic of discussion in SA procedures. The QoNM procedure is a voluntary pathway towards regulatory qualification of methodologies in drug development, which also includes biomarkers, and can result in a Qualification Advice (QA) or Qualification Opinion (QO) (Figure 1) (13). A QA typically concerns projects in earlier stages, potentially including review of preliminary data, and is the way to agree on evidence generation plans and protocols for studies intended to support a QO. When the submitted evidence supports a QO, the draft opinion document is published for consultation by the scientific community before final adoption by EMA’s Committee for Medicinal Products for Human Use (CHMP). Upon final adoption of the QO, it is considered that the proposed method (e.g., biomarker) is an acceptable regulatory standard for the defined context of use in drug development. By making the QO publicly available, others may use the qualified method or biomarker in their drug development efforts. In this study, we analysed the journey of biomarkers through the EMA’s pre-submission interaction platforms and beyond, by reviewing interactions between developers and the Agency as well as the use of qualified biomarkers in clinical trials. In doing so, we aimed to identify potential points for improvement, with the goal to enhance the seamless support by the EMA for biomarker validation, qualification, and subsequent use in drug development.

## Methods

### Search and Analysis of Innovation Task Force Briefing Meetings

Minutes from ITF briefing meetings that took place between January 1st, 2008 and December 31st, 2020 were collected from EMA’s internal database. The most recent version of the minutes file was used and, wherever possible, the final version reviewed and approved by ITF and participants. Minutes of preliminary meetings or informal teleconferences were excluded from the analysis. This collection was searched using keywords *biomarker*, *in vitro, companion, diagnostic* and *qualification*. Initially, all documents were selected that (1) included the word *biomarker*, (2) contained the word *companion* if it occurred together with the term *diagnostic* or *test*, or (3) contained the word *qualification* in combination with *procedure*, *advice*, or *novel methodologies*. Minutes that referred to both biomarkers and QoNM (1), minutes that referred to the use of *in vitro* diagnostics in combination with biomarkers (2), and minutes in which biomarkers were the main topic of discussion (3) were marked as relevant. Related fee-associated procedures were identified as follows: for QoNM, applicant names and relevant keywords from the ITF minutes were used to search a collection of biomarker-related QoNM procedures that took place between 2008 and 2020 (described in the next section) as well as QoNM applications that had been withdrawn or rejected. Similarly, the applicant’s name and relevant keywords (including *biomarker*) were used to search all finished SA procedures that had been started in the year of the ITF meeting or later. Hits from these searches were inspected manually and those that discussed the biomarker from the ITF meeting were marked as relevant.

### Search and Analysis of Qualification of Novel Methodologies Procedures

A document containing a list of all QoNM procedure applications was downloaded from EMA’s internal database on May 11th, 2021. Procedures that never started, or that were started after December 31st, 2020, were excluded from the analysis. All remaining procedures were assessed individually and procedures that contained modelling or simulation techniques, patient-reported outcomes, ratings or scales, methods or protocols, clinical outcome assessments, or databases or registries were excluded from the analysis.

### Clinical Trials Search

Clinical trial searches were performed in the ClinicalTrials.gov database using the general search function or the expert search function (Supplementary Table 1). The respective disease areas of the qualified biomarkers were searched for interventional trials in which the biomarker in question was used according to the context of use endorsed by the CHMP in the qualification opinion (Table 1). For each search, all hits were downloaded, and data were extracted, including NTC number, title, status, condition, outcome measures, sponsors, total number, and age range of enrolled subjects, start and completion date, and locations of the trial. As most qualified biomarkers serve the purpose of enrichment of study populations, the “inclusion criteria” and “exclusion criteria” sections were manually extracted for each trial. The relevant sections were screened individually to determine whether the search terms occurred in the desired context, e.g., whether the biomarker was in fact used for enrichment and if so, what cut-off values were used. Based on this exercise, several initial hits were deemed irrelevant and were excluded from further analysis.

**TABLE 1:** Qualification opinions related to biomarkers.

Published	Biomarker and Context of Use	Related SA/QoNM	Clinical Trials
October 2010	ILSI/HESI Novel Renal Toxicity Biomarkers	None	Not for clinical use
April 2011	Low Aβ 1-42 and high tau is qualified as a predictive (prognostic?) marker for an evolution to dementia in patients diagnosed with mild cognitive impairment. The ratio is discussed but not qualified, no cut-off values are qualified.	SA in 2009 and 2011 Follow up QO procedures in 2011 (2)	28 hits, 26 after QO
November 2011	Low hippocampal volume, as measured by MRI and considered as a dichotomised variable (low volume or not), might be considered a (prognostic) marker of progression to dementia in subjects with cognitive deficit compatible with predementia stage of AD. No cut-off value has been qualified.	SA in 2011, 2012 and 2019 Previous QA procedure in 2010	4 hits, all after QO
February 2012	Positive amyloid PET signal qualifies to identify patients with clinical diagnosis of predementia AD who are at increased risk to have an underlying AD neuropathology, for the purpose of enriching a clinical trial population.	SA in 2012 and 2014 Previous QO procedure in 2010, follow up QO procedure started in 2011	120 hits
February 2012	CSF biomarker signature based on low Aβ 1-42 and high T-tau as well as a positive amyloid PET signal qualify to identify patients with clinical diagnosis of mild to moderate AD who are at increased risk to have an underlying AD neuropathology, for the purpose of enriching a clinical trial population.	SA in 2009 and 2011	PET AND amyloid: 120 PET OR CSF: 12
October 2015	Baseline total kidney volume, in combination with patient age and eGFR, as a prognostic biomarker to identify patients with Autosomal Dominant Polycystic Kidney Disease that are likely to experience a progressive decline in renal function.	SA in 2009 and 2011	8 hits, 4 after QO
April 2018	Changes in plasma fibrinogen levels as a prognostic biomarker in chronic obstructive pulmonary disease. The threshold that is considered most useful is 350mg/dl.	SA in 2016	3 hits, 1 after QO
April 2018	Dopamine Transporter levels by SPECT Neuroimaging as an enrichment biomarker for clinical trials targeting patients with early Parkinsonian symptoms.	Previous QA procedures in 2015 and 2016	14 hits, 7 after QO
April 2019	Stride velocity 95th centile (SV95C) measured at the ankle is an acceptable secondary endpoint in clinical trials for ambulant Duchenne Muscular Dystrophy (DMD) patients 5 years of age and above.	SA in 2020	2 hits, 1 after QO

## Results

### Majority of Applicants Do Not Engage in Fee-Related Procedures After Innovation Task Force Briefing Meetings

**FIGURE 2:**
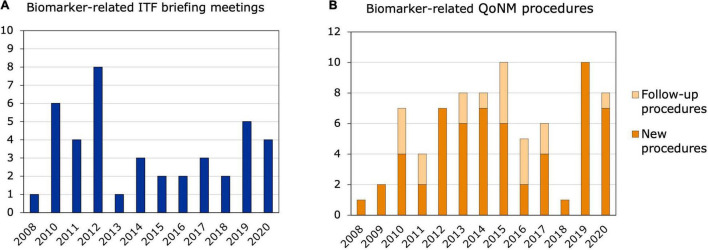
Biomarker-related interactions between 2008 and 2020. **(A)** ITF briefing meetings. The bars represent ITF briefing meetings related to biomarkers and their qualification that took place from 2008 to 2020. **(B)** New and follow-up qualification procedures. Qualification of Novel Methodologies procedures related to biomarkers that were started between 2008 and 2020.

Out of the 311 ITF briefing meetings that took place from 2008 to 2020, 41 contained discussions or questions related to biomarkers and, in most cases, applicants were referred to SA or QoNM. Most biomarker-related ITF meetings were held in 2010 and 2012, with six and eight meetings, respectively, but no increasing trend was observed (Figure 2A). At least 12 of the 41 meetings could be linked to relevant SA or QoNM procedures (Supplementary Table 2). Six ITF meetings were linked to a SA procedure that referred to the same biomarker. For example, a lung clearance index that was discussed in an ITF meeting as a potential surrogate endpoint in cystic fibrosis trials was endorsed by the CHMP as a primary endpoint in the SA procedure. In another ITF meeting, a predictive biomarker for patient selection in non-small cell lung cancer trials was discussed. Two related SA procedures were identified that contained discussions on the cut-off values for that biomarker. In yet another ITF meeting, predictive biomarkers for clinical trials in multiple sclerosis were discussed, which could also be linked to a SA procedure. Interestingly, no reference was made to QoNM in any of the final SA letters. Seven ITF meetings resulted in follow-up QoNM procedures, one of which was also linked to a SA procedure. Four of the seven ITF meetings were with consortia funded by the Innovative Medicines Initiative (IMI): an EU public-private partnership funding health research and innovation (14, 15).

### Consortia Are More Likely to Request Follow-Up Advice to Previous Qualification of Novel Methodologies Procedures

Out of the 77 biomarker-related qualification procedures that took place between 2008 and 2020, 18 were follow-ups to previous procedures (Figure 2B). Nine of the 77 procedures resulted in a QO, of which four were follow-up procedures (Table 1). Two QOs from 2011 (16, 17) were follow-ups to the same QO procedure in 2010 (18) and qualified biomarkers for Alzheimer’s Disease (AD). These three QOs are the only QO procedures that were brought forward by a company and aimed at qualifying a biomarker for a specific clinical development program. All three qualified biomarkers are enrichment biomarkers for clinical trials with BMS-708163, or avagacestat: an amyloid precursor protein secretase (γ-secretase) inhibitor that was developed by Bristol-Myers Squibb for the treatment of predementia and mild-to-moderate AD. Another QO, which was a follow-up to previous QA procedures, qualified low hippocampal volume as an enrichment biomarker for clinical trials in predementia AD (19). The fourth follow-up QO qualified neuroimaging of the dopamine transporter as an enrichment biomarker for Parkinson’s Disease (PD) (20) and was preceded by two previous QA procedures—an initial QA procedure and a follow-up QA. Of all biomarker-related qualification procedures, 36 were started by companies, of which 20 related to a specific clinical development program. On the other hand, 41 procedures were initiated by consortia or foundations and the vast majority of those by IMI consortia and the Critical Path Institute (C-Path). The C-Path initiative is a non-profit public-private partnership with the FDA, which aims to accelerate the pace and reduce the costs of medical product development through the creation of new standards, including biomarkers, that aid in the scientific evaluation of the efficacy and safety of new therapies (21, 22). Interestingly, only three out of the 18 follow-up procedures were initiated by companies, and the remaining 15 by consortia or foundations. Among the follow-up procedures, a large number related to safety biomarkers for drug-induced injury in different organ systems: five of them in the kidney, but also in the liver and cardiovascular system. These procedures were also mainly driven by the C-Path Preclinical Safety Testing Consortium and the IMI SAFE-T consortium. The IMI EU-AIMS consortium also initiated three follow-up QAs in autism spectrum disorder (23).

### Context of Use Endorsed in Qualification Opinion Is Not Always Respected

Since 2008, eight qualification opinions on biomarkers for clinical use have been adopted by the EMA (Table 1). The first four of these concern prognostic/predictive biomarkers for enrichment in clinical trials in AD and were published between April 2011 and February 2012. This includes the three procedures initiated by Bristol-Myers Squibb for the clinical development of avagacestat, which was discontinued in November 2012 as the Phase II clinical trial programme did not establish the desired efficacy profile. The three QOs qualify cerebrospinal fluid (CSF) biomarkers “*low A*β*1-42 and high tau concentrations*” as well as “*a positive amyloid PET signal*” for enrichment in clinical trials for predementia AD and mild to moderate AD (16–18, 24). The company performed two Phase II studies with avagacestat (NCT00890890 and NCT00810147), only one of which used inclusion criteria related to the QOs. This study was started in 2009, before the first qualification procedure, and “*CSF a*β*42 levels* < *200 pg/mL or Total Tau/a*β*42 ratio of* ≥ *0.39*” were used as inclusion criteria. A search of the clinicaltrials.gov database for trials using amyloid beta and/or tau in their inclusion criteria yielded 28 relevant hits, two of which were started before publication of the opinions (Table 2). Interestingly, the qualified biomarker “*low A*β*1-42 and high tau*” is used in only six out of 28 trials. Although the QO does not specify what would be considered “low” and “high” values, two trials mention similar cut-offs: “A-beta 42 concentration of less than 638 ng/L AND total tau >375 ng/L” (NCT02389413) and “low Aβ1-42 concentrations (<640 pg/mL) and increased total tau concentrations (>375 pg/ml)” (NCT02240693). In several cases, “low Aβ1-42” or “high tau” are used, but not in the right combination. Another observation is that the ratio between Aβ1-42 and either total tau, tau, or phosphorylated tau is mentioned as a biomarker in 13 trials, with varying thresholds. A total of 12 out of 28 trials also mention the qualified biomarker “positive amyloid PET scan” as part of their inclusion criteria, however, always as an alternative to CSF biomarkers and never a combination of the two. A search of the clinicaltrials.gov database for trials using “amyloid AND PET” in their inclusion criteria yielded 120 relevant hits, 12 of which are included in Table 2. The fourth procedure related to AD qualified low hippocampal volume, as measured by MRI, and considered as a dichotomised variable (low volume or not), as a prognostic marker of progression to dementia in subjects with cognitive deficit compatible with predementia stage of AD (19, 25). Like for the CSF biomarkers, no cut-off value for “low volume” was mentioned in the QO. A search of the clinicaltrials.gov database for trials using “hippocampal volume” in their inclusion criteria yielded 4 hits in total, all of which were started after publication of the QO. Besides the trial included in Table 2, which mentions “hippocampal volume loss,” the other three contain the following inclusion criteria:

**TABLE 2:** Use of CSF biomarkers for enrichment of clinical trials in AD.

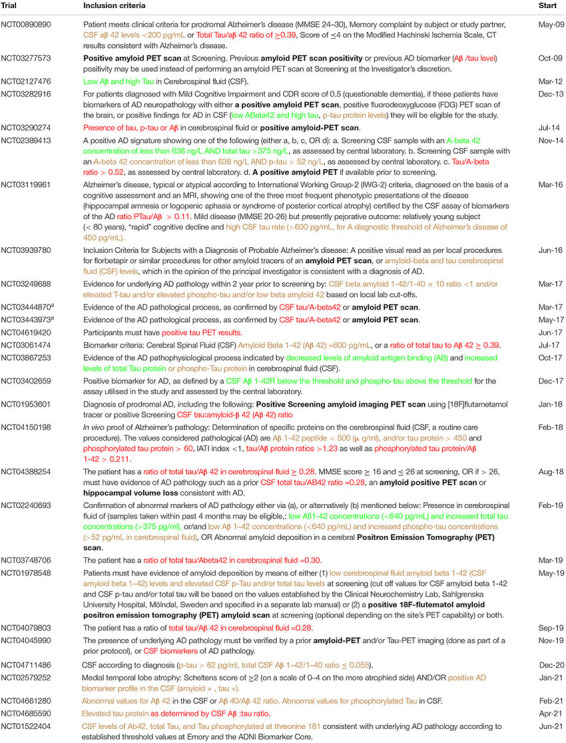

*Trials were retrieved by searching all interventional trials in the clinicaltrials.gov database using search strings high tau, tau AND ratio, and amyloid AND PET in inclusion criteria, in trials for Alzheimer’s Disease and without date restrictions. The relevant sections of the inclusion criteria have been coloured as follows: use of biomarkers as qualified, i.e., “high tau and low Aβ1-42” (green), use of either one of the criteria, i.e., “high tau and/or low Aβ1-42” or use of phosphorylated tau (orange), and use of presence of, or a ratio between (p-)tau and Aβ1-42 (red). The use of other qualified biomarkers, such as positive amyloid PET scan or hippocampal volume, has been marked in bold.*

“*Magnetic resonance imaging (MRI) scans of the brain within the past six months reveal evidence and findings consistent with Alzheimer’s disease, including hippocampal volume loss and/or overall cerebral atrophy (cerebral volume loss).”*

“*Magnetic resonance imaging (MRI) confirmation of atrophy of the hippocampus or the medial temporal lobe volume, MRI manifestation of high possibility of Alzheimer’s Diseas*e.”

“*Magnetic Resonance Imaging (MRI) confirmation of atrophy of the hippocampus or the medial temporal lobe volume.”*

The remaining four QOs regarding biomarkers for clinical use were published between 2015 and 2020. In October 2015, baseline total kidney volume (TKV) was qualified as a prognostic biomarker for renal decline in autosomal dominant polycystic kidney disease (26, 27). The QO mentions that baseline TKV should be used in combination with other markers, e.g., patient age and eGFR, to identify patients that are likely to experience a progressive decline in renal function. A search of clinicaltrials.gov for interventional trials in kidney disease yielded a total of eight trials, four of which started after the publication of the QO (Supplementary Table 3). Six of the eight trials refer to the “total” or “combined” kidney volume, of which five refer to a specific value that ranges from >500 to > 1,200 mL. Kidney volume progression, specifically with a yearly increase of more than 6%, is also mentioned as an inclusion criterium in three trials. None of the trials use TKV in combination with other factors.

In April 2018, changes in plasma fibrinogen levels were qualified as a prognostic biomarker in chronic obstructive pulmonary disease, with 350 mg/dl as the threshold considered to be most useful (28, 29). A search of clinicaltrials.gov yielded three hits, only one of which was started after publication of the QO (Supplementary Table 4). All three trials referred to plasma fibrinogen concentration at baseline, rather than changes in levels: two trials referred to a threshold of 350 mg/dL and the third to 300 mg/dL.

In April 2018, reduced dopamine transporter (DAT) levels, as measured by Single-Photon Emission Computed Tomography (SPECT) neuroimaging were qualified as an enrichment biomarker for clinical trials targeting patients with early Parkinsonian symptoms (20, 30). The qualification procedure was initiated by the Critical Path for Parkinson’s consortium with the aim to demonstrate the predictive accuracy of visual assessment of DAT neuroimaging scans at baseline for identifying those subjects with high likelihood of progressing in clinical motor disability. A search of clinicaltrials.gov yielded 14 relevant hits, half of which were started after publication of the QO (Supplementary Table 5). Two out of the trials used DAT SPECT as means of exclusion.

In April 2019, stride velocity 95th centile (SV95C) measured at the ankle was qualified as a secondary endpoint in clinical trials for ambulant Duchenne Muscular Dystrophy patients of 5 years or older (31, 32). From the adopted QO document: “Stride velocity 95th centile measured at the ankle (SV95C) is an acceptable secondary endpoint in pivotal or exploratory drug therapeutic studies for regulatory purposes when measured by a valid and suitable wearable device to quantify a patient’s ambulation ability directly and reliably in a continuous manner in a home environment and as an indicator of maximal performance.” A search of clinicaltrials.gov yielded two hits by different sponsors, one of which started after publication of the QO (Supplementary Table 6).

## Discussion

Support for biomarker discovery, validation, and qualification is an important objective at the European Medicines Agency (8, 9). Biomarker developers can make use of various interaction platforms offered by the Agency, ranging from briefing meetings with the Innovation Task Force as a first point of contact, to the Qualification of Novel Methodologies procedure, where a biomarker can be qualified for a specific context of use (13). Since the start of this voluntary procedure in 2008, nine biomarkers have obtained this regulatory “stamp of approval” by the EMA, which has sparked the question how the support from the regulatory system for biomarker qualification can be improved. The aim is to enable precision medicine for the benefit of patients, by facilitating a seamless interaction with biomarker and medicine developers. Encouraged by this objective, we reviewed biomarker-related pre-authorisation interactions that took place between 2008 and 2020 at the EMA and explored the impact of qualified biomarkers, by assessing their uptake in clinical trials.

Of the 41 biomarker-related ITF briefing meetings that were identified between 2008 and 2020, 12 could be linked to interactions between the applicant and the EMA through Scientific Advice or QoNM. The fact that 70% of meetings did not result in any other interaction might be due to the early stage of some projects, or the fees associated with the abovementioned procedures. A decreasing trend in the use of QoNM by pharmaceutical companies had previously been identified (33), suggesting that companies are more likely to include biomarker-related questions in SA procedures. This hypothesis is supported by the data on interactions following ITF briefing meetings, which show that half of the applicants that initiated a follow-up opted for SA, all of which were companies, when they had been referred to the qualification procedure by ITF experts. On the other hand, most ITF meetings that resulted in QoNM were with consortia funded by the Innovative Medicines Initiative, which was launched in 2008 to address challenges in drug development and regulation (14, 15). Many IMI projects generate data that is relevant for stakeholders in medicines development and, therefore, involvement of regulators is a cornerstone of the IMI programme. Yet, despite various IMI projects aiming at biomarker qualification, none of them have resulted in qualified biomarkers thus far. This could be due to the limited timespan of the IMI-funded projects, typically 5–6 years, which may be too short for complex biomarker validation exercises. This also became evident from analysis of QA final advice letters and is in line with what has been reported by Laverty and Meulien, who state that the interaction with regulatory bodies is often initiated too late in the project (15). However, consortia such as IMI EU-AIMS are mentioned as a success story due to their early interaction with regulators, which has resulted in multiple follow up QA procedures in the field of autism spectrum disorder (23). These findings highlight that the benefit of transparency and availability of biomarker qualification data should be communicated clearly, in order to engage a wide range of stakeholders in this procedure.

The difference between the number of ITF meetings in which QoNM was recommended and the number of applicants that follow this recommendation suggests that QoNM application may be perceived as challenging. Given that applicants are encouraged to make use of this procedure early on in their biomarker qualification effort, and that the interactions should ideally follow an iterative approach (34), lowering potential hurdles is key to achieving these aims. As part of the QoNM procedure, applicants can participate in a *preparatory meeting* with the Scientific Advice Office, in which members of the qualification team may join as appropriate (34). Such meetings, which take place before any fee is due, allow for an informal scientific discussion and may offer preliminary feedback on the maturity of the data. However, preparatory meetings take place only after submission of a complete draft dossier for assessment, which may discourage applicants, particularly in early stages of a project. In such cases, questions around the qualification procedure and the level of evidence required for biomarker qualification are often addressed in ITF briefing meetings. To facilitate access to the QoNM procedure, beyond the information currently available (35, 36), additional guidance may aid applicants in preparation of the draft dossier, especially in early-stage projects. Further support can be obtained through informal interactions with the EMA Scientific Advice Office (scientificadvice@ema.europa.eu).

To investigate some aspects of the potential impact of a QO on medicines development, the use of qualified biomarkers in clinical trials was assessed. A search of the clinicaltrials.gov database revealed that the first qualified biomarkers, CSF proteins (sometimes combined with amyloid PET) as enrichment biomarkers for AD, were used as inclusion criteria in 26 trials after publication of the opinion. However, an investigation of the inclusion criteria showed that only six trials used the biomarkers according to the qualified context of use (“high tau and low Aβ1-42”). It is unclear what exactly is meant by “high” and “low” tau, which has also been discussed in the comments from the public consultation (37). The same applies to the QOs on Low Hippocampal Volume, Total Kidney Volume, and Plasma Fibrinogen levels—a threshold that was considered “most useful” was only given for TKV. These findings highlight the importance of a clearly defined context of use, in order to ensure optimal use of the qualified biomarker. Overall, qualified biomarkers are used in clinical trials, albeit not always according to their qualified context of use. This observation is also supported by findings from SA procedures, where sponsors often refer to specific qualified biomarkers but want to use the biomarker in a different context of use (data not shown). It should be noted that a biomarker may be scientifically valid in different contexts of use, which may not all be covered in a QO procedure. In general, as clinical trials are the foundation of evidence generation for MAAs, the uptake of qualified biomarkers highlights that regulatory qualification is relevant for medicine developers.

In summary, regulatory qualification of biomarkers is a cornerstone of the EMA’s strategy to enable precision medicine for the benefit of patients. This study presents a review of biomarker-related pre-authorisation interactions at the EMA since 2008, which highlights opportunities to enhance the seamless support for biomarker developers. More detailed guidance may facilitate QoNM application for applicants that are referred to the qualification procedure during ITF briefing meetings, enabling sponsors to engage in preparatory meetings with members of the Qualification Team. Moreover, early initiation of dialogue with regulators is key to successful biomarker qualification by consortia, such as those initiated by the IMI initiative or CriticalPath Institute. In general, the use of qualified biomarkers in clinical trials illustrates the positive impact of regulatory qualification on evidence generation for MAAs. However, a review of inclusion criteria and outcome measures of the trials showed that, although the biomarker may be scientifically valid for the intended purpose, the context of use endorsed by the CHMP is not always applied. An assessment of the impact on MAA evaluation may contribute to understanding the value of a QO and may encourage potential applicants to engage in the procedure, which in turn would contribute to the development of precision medicine.

## Data Availability Statement

The original contributions presented in the study are included in the article and Supplementary Material. Further inquiries can be directed to the corresponding authors.

## Author Contributions

NH, FE, JL, and AH designed the study. NH performed the research and analysed the data. NH, FE, and TV wrote the manuscript. All authors contributed to the article and approved the submitted version.

## Author Disclaimer

The views expressed in this article are the personal views of the authors and may not be understood or quoted as being made on behalf of or reflecting the position of the European Medicines Agency or one of its committees or working parties.

## Conflict of Interest

The authors declare that the research was conducted in the absence of any commercial or financial relationships that could be construed as a potential conflict of interest.

## Publisher’s Note

All claims expressed in this article are solely those of the authors and do not necessarily represent those of their affiliated organizations, or those of the publisher, the editors and the reviewers. Any product that may be evaluated in this article, or claim that may be made by its manufacturer, is not guaranteed or endorsed by the publisher.
