# Supplementary material for: Biomarkers in Medicines Development—From Discovery to Regulatory Qualification and Beyond
Source: Front Med (Lausanne). 2022 Apr 26;9:878942. doi: 10.3389/fmed.2022.878942 (PMC9086587; doi:10.3389/fmed.2022.878942)
Supplement: Supplementary file 1 [file Data_Sheet_1.PDF]

Supplementary Table 1: Overview of clinical trials searches performed on ClinicalTrials.gov.

Search	Search string	Total hits	Comments
1	Expert search: AREA[EligibilityCriteria] **high tau** AND AREA[StudyType] EXPAND[Term] COVER[FullMatch] "Interventional" AND AREA[ConditionSearch] Alzheimer disease	21	12 relevant hits
2	General search: drug name BMS-708163	13	1 relevant hit
3	Expert search: AREA[EligibilityCriteria] **(amyloid AND PET)** AND AREA[StudyType] EXPAND[Term] COVER[FullMatch] "Interventional" AND AREA[ConditionSearch] Alzheimer disease	164	120 relevant hits
4	Expert search: AREA[EligibilityCriteria] **(tau AND ratio)** AND AREA[StudyType] EXPAND[Term] COVER[FullMatch] "Interventional" AND AREA[ConditionSearch] Alzheimer disease	15	6 new hits, all relevant
5	Expert search: AREA[EligibilityCriteria] **hippocampal volume** AND AREA[StudyType] EXPAND[Term] COVER[FullMatch] "Interventional" AND AREA[ConditionSearch] Alzheimer disease	4	3 new hits, all relevant
6	AREA[EligibilityCriteria] **"kidney volume"** AND AREA[StudyType] EXPAND[Term] COVER[FullMatch] "Interventional" AND AREA[ConditionSearch] **kidney disease**	8	8 relevant hits
8	AREA[EligibilityCriteria] **"plasma fibrinogen"** AND AREA[StudyType] EXPAND[Term] COVER[FullMatch] "Interventional" AND AREA[ConditionSearch] **chronic obstructive pulmonary disease**	3	3 relevant hits
9	AREA[EligibilityCriteria] **(("dopamine transporter" OR "DAT") AND "SPECT")** AND AREA[StudyType] EXPAND[Term] COVER[FullMatch] "Interventional" AND AREA[ConditionSearch] **parkinson's disease**	16	16 relevant hits
10	AREA[OutcomeSearch] **("stride velocity" OR "actimyo")** AND AREA[StudyType] EXPAND[Term] COVER[FullMatch] "Interventional" AND AREA[ConditionSearch] **Duchenne**	2	2 relevant hits
11	General search for **("stride velocity" OR "actimyo")** in interventional trials in **Duchenne Muscular Dystrophy**	2	Same 2 as above

Supplementary Table 2: ITF meetings with follow up in form of Scientific Advice (SA) or Qualification Advice (QA).

ITF meeting	Description	Follow-up
March 2010	Exploratory-phase qualification of clinical Drug-Induced Liver Injury (DILI) biomarkers (safety, monitoring, diagnostic, prognostic)	QA (2013), FU-QA (2013)
June 2010	General discussion on PD/Response biomarkers for clinical trials CNTO136 programme in Rheumatoid Arthritis.	SA: 3 hits, but no mention of the biomarker
June 2010	Exploratory-phase qualification of clinical Drug-Induced Kidney Injury (DIKI) biomarkers (safety, monitoring, diagnostic, prognostic)	QA (2013), FU-QA (2013), FU-QA (2014), FU-QA (2016)
October 2010	Exploratory-phase qualification of clinical Drug-Induced Vascular Injury (DIVI) biomarkers (safety, monitoring)	QA (2013), FU-QA (2016)
March 2011	Periostin as predictive biomarker to identify patients with severe asthma that may benefit from blockade of IL13 by Lebrikizumab	QA (2012), SA (2015)
March 2011	Predictive biomarker for selection of patients with “Met-positive” non-small cell lung cancer for trials	SA (2011), SA (2012)
September 2012	General discussion on predictive biomarkers for clinical trials in MS	SA (2012)
September 2012	Classification of cystic fibrosis patients by predictive biomarker CTFR mutations/molecular phenotypes	SA (2017)
December 2012	Lung Clearance Index as a surrogate endpoint in cystic fibrosis trials	AS (2017)
October 2014	General discussion on biomarkers for clinical trials in type 1 diabetes	QA (2019): not on biomarkers, but on trial protocol design
October 2015	Genomic Allergen Rapid Detection (GARD): predictive biomarker signature as screening tool in early drug discovery	Applied for QOQA in December 2015, withdrawn
October 2018	Biomarkers for non-alcoholic fatty liver disease and non-alcoholic steatohepatitis (Diagnostic, Prognostic, Monitoring, PD/Response)	QA (2019), QA (2019)

Supplementary Table 3: Total kidney volume as an inclusion criterium in clinical trials. Trials were retrieved by searching all interventional trials in the clinicaltrials.gov database using search string “kidney volume” in inclusion criteria, in trials for kidney disease and without date restrictions. The relevant sections have been marked in bold.

Trial	Inclusion criteria	Start
NCT02115659	Documented **kidney volume progression** with yearly **increasing rate more than 6%**	Jun-06
NCT00346918	Documented **kidney volume progression**	Jun-09
NCT04578548	Rapidly progressive disease, defined as presence of all of the following: **Total Kidney Volume (TKV) >750 mL**, as determined on imaging not older than 5 years before screening.	Dec-10
NCT00920309	**Combined kidney volume >1200 ml**	Apr-14
NCT01233869	**Total kidney volume ≥ 750 cc**, as measured by centrally evaluated MRI.	Sep-16
NCT02948179	The patient with **total kidney volume more than 650ml** OR the patient with **total kidney volume increase rate more than 6% every year**	Aug-17
NCT03523728	Mayo Imaging Classification of ADPKD Class 1C, 1D or 1E: **Total kidney volume (TKV) must be confirmed** by a central reader prior to Visit 3.	Oct-18
NCT03273413	**Total kidney volume >500 mL**	Nov-20

Supplementary Table 4: Plasma fibrinogen levels as an inclusion criterium in clinical trials. Trials were retrieved by searching all interventional trials in the clinicaltrials.gov database using search string "plasma fibrinogen" in inclusion criteria, in trials for COPD and without date restrictions. The relevant sections have been marked in bold.

Trial	Inclusion criteria	Start
NCT04268823	Patients with **plasma fibrinogen level ≥ 350 mg/dL** at screening.	Feb-14
NCT03034967	Participants with a documented history of COPD exacerbation(s) in the 12 months prior to study participation (screening) meeting at least one of the following criteria: >=2 COPD exacerbations resulting in prescription for antibiotics and/or oral corticosteroids or hospitalization or extended observation in a hospital emergency room or outpatient center; 1 COPD exacerbation resulting in prescription for antibiotics and/or oral corticosteroids of hospitalization or extended observation in a hospital emergency room or outpatient center and a **plasma fibrinogen concentration at screening >=3 grams/liter (300 milligram/deciliter).**	Apr-17
NCT02130193	Subjects with a documented history of COPD exacerbation(s) in the 12 months prior to study participation meeting at least one of the following criteria: >=2 COPD exacerbations resulting in prescription for antibiotics and/or oral corticosteroids or hospitalization or extended observation in a hospital emergency room or outpatient center; 1 COPD exacerbation resulting in prescription for antibiotics and/or oral corticosteroids or hospitalization or extended observation in a hospital emergency room or outpatient center and a **plasma fibrinogen concentration at screening >=3.5 milligram/milliliter (mg/mL).**	Sep-20

Supplementary Table 5: Levels of dopamine transporter as enrichment biomarker in Parkinson's Disease. Trials were retrieved by searching all interventional trials in the clinicaltrials.gov database using search string (("dopamine transporter" OR "DAT") AND "SPECT") in inclusion criteria, in trials for PD and without date restrictions. The relevant sections have been marked in bold.

Trial	Inclusion criteria	Start
NCT04284436	**Positive DaTscan™ SPECT** by quantitative readout for idiopathic Parkinson disease.	Apr-00
NCT03710707	Screening dopamine transporter (DAT) SPECT scan with a **DAT deficit consistent with Parkinson's disease**	Jun-10
NCT03716570	Screening **dopamine transporter (DaT)/ single-photon emission computed tomography (SPECT) results consistent with neurodegenerative Parkinsonism** (central reader).	Feb-12
NCT04777331	Dopamine transporter imaging with single photon emission computed tomography **(DaT-SPECT) imaging consistent with dopamine transporter deficit**, as assessed by the central reader	Dec-14
NCT03318523	Screening **dopamine transporter (DaT)/ single-photon emission computed tomography (SPECT) results consistent with neurodegenerative Parkinsonism** (central reading).	Oct-16
NCT01141023	Confirmation from imaging core that screening **DAT scan is consistent with dopamine transporter deficit**, or if applicable a VMAT-2 PET scan consistent with vesicular monoamine transporter deficit.	Jun-17
NCT04658186	A Screening Dopamine Transporter Imaging with Single Photon Emission Computed Tomography **(DaT-SPECT) is consistent with PD** as determined by a central reader	Jan-18
NCT04691661	**Positive DAT-scan (e.g. a striatal dopamine transporter deficit on dopamine transporter imaging by DaT-SPECT**, characterized by crescent-shaped areas of asymmetrical aspect, or of symmetrical aspect but of uneven intensity, between the right and the left brain hemisphere) confirmed by local reading;	May-18
NCT01568099	Brain magnetic resonance imaging (MRI) scan and **DAT-SPECT scan are consistent with the diagnosis of PD**	Feb-19
NCT03587649	Have screening or prior **DaTscan SPECT imaging demonstrating evidence of dopamine transporter deficit** based on visual read.	Aug-19
NCT04075318	Dopamine transporter single-photon emission computerized tomography scan (DaTscan) **inconsistent with dopamine transporter deficit** (i.e. exclusion).	Dec-20
NCT03655236	Exclusion: Most recent **DaT SPECT scan not compatible with PD** (i.e., **Scans Without Evidence of Dopaminergic Deficit** [SWEDD]) based on central read by a study physician;	Mar-21
NCT03100149	A screening brain **DaT-SPECT consistent with PD** (central reading)	Mar-21
NCT02267434	The result of a **DAT-SPECT** and MRI examination of the patient's brain has to be **consistent with the diagnosis of PD**	May-21

Supplementary Table 6: Stride velocity 95th centile as outcome measure in clinical trials for Duchenne Muscular Dystrophy. Trials were retrieved by searching all interventional trials in the clinicaltrials.gov database using search string ("stride velocity" OR "actimyo"), in trials for DMD and without date restrictions. The relevant sections have been marked in bold.

Trial	Outcome measures	Start
NCT03039686	Secondary outcome measure: **Change from Baseline** at Week 48 in **95th Percentile Stride Velocity** [Time Frame: Baseline, Week 48]. Stride velocity was recorded with the ActiMyo device in a subset of the overall study population. The ActiMyo device measures the daily movement and activity levels of the participant. The device consists of two sensors worn on each ankle. A higher velocity reflects a better outcome. A positive change from baseline indicates an improvement.	Jul-17
NCT03907072	Secondary outcome measure**: Change from baseline in the 95th percentile of stride velocity** [Time Frame: Day 1 through Week 48]	Sep-19
